# Dynamic Cervical Cord Compression Post-laminectomy Visualized by Flexion-extension Magnetic Resonance Imaging: Case Report

**DOI:** 10.7759/cureus.3878

**Published:** 2019-01-14

**Authors:** Adam Y Li, Jennifer B Dai, Alexander F Post, Tanvir F Choudhri

**Affiliations:** 1 Neurosurgery, The Icahn School of Medicine at Mount Sinai, New York, USA

**Keywords:** cervical laminectomy, complication, cord compression, flexion/extension, paraspinal muscle, mri

## Abstract

Flexion-extension magnetic resonance imaging (MRI) in the cervical spine is not universally used in cervical spine surgery. However, flexion-extension MRIs can identify previously undetected spinal stenosis, improve surgical decision-making, and maybe a better tool to evaluate postoperative outcomes. One uncommon complication after laminectomy, to treat cervical spinal stenosis, is muscle weakness due to subsequent dynamic cord compression by posterior paraspinal musculature. We present a case of a 41-year-old male who underwent posterior cervical decompression and developed subsequent neurological deficits and muscle weakness. MRI with neutral cervical positioning did not show spinal stenosis necessitating surgical intervention. However, given the patient’s increasing tetraparesis, flexion-extension MRI was performed and it revealed significant spinal stenosis in both flexion and extension positions due to spondylosis and compression from paraspinal muscles. This case demonstrates the utility of flexion-extension MRI in identifying pathologies such as cord compression by paraspinal muscles. Exclusive use of a neutral-position MRI scan may not be sufficient to provide proper diagnoses for cervical spine pathologies. Flexion-extension MRI should be considered when the degree of neurological symptoms outweighs minimal or absent pathology seen on neutral-position sagittal MRI.

## Introduction

The cervical spine is highly mobile with complex kinematics, and neck motions require varied coordinated movements between individual cervical vertebrae. When injury or disease occurs, cervical spine movements become more difficult to understand, and extensive research has been done to study cervical spine movements after injury from contact sports, whiplash, pressure gradients, and other causes [1]. Despite this complexity, computed tomography (CT) and magnetic resonance imaging (MRI) of cervical spine injuries typically use a static, uniform anatomical position, which is unable to capture the full complexity of motion and disease in the cervical spine. This can be partially remedied by flexing and extending the cervical spine during imaging, and flexion-extension MRI has been used for various types of pathologies including spinal stenosis [2-9], rheumatoid arthritis [10], trauma [11], Down syndrome [12], and Chiari I malformation [13].

In the cases of cervical spinal stenosis, significant changes in intervertebral disc bulge, ligamentum flavum bulge, size of the spinal canal, cord diameter, and cord length have been reported during flexion-extension MRI, requiring surgical intervention in symptomatic cases with cord compression. When evaluating postoperative outcomes, flexion-extension MRI can help identify complications not seen in a neutral position. One uncommon complication better visualized by dynamic MRI is post-laminectomy cord compression by paraspinal muscles. Only 13 cases have been previously reported, and all require additional decompressive surgery due to compression during cervical extension [4, 14-17]. This report presents a case where cervical MRI post-laminectomy and discectomy in a neutral position did not show significant cord compression despite postoperative neurological symptoms. A flexion-extension MRI was performed, and found increased cord compression for both flexion and extension positioning due to residual spondylosis and posterior compression by paraspinal muscles, indicating a need for additional decompressive surgery.

## Case presentation

Clinical history

**Figure 1:**
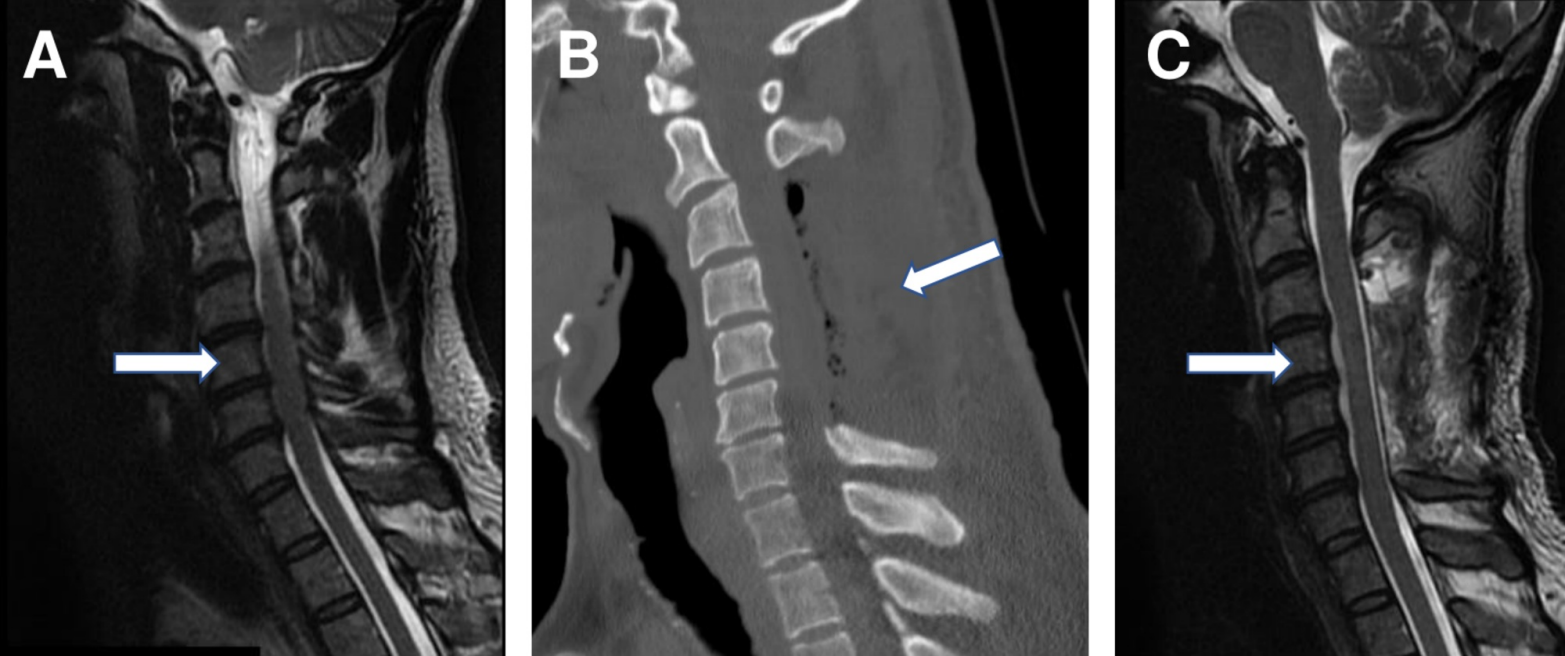
Neutral Position Preoperative and Postoperative Imaging of the Cervical Spine Preoperative sagittal T2 fast recovery fast spin echo magnetic resonance imaging (A) shows cervical spinal stenosis requiring decompression. Postoperative sagittal computed tomography (B) after C3-C6 laminectomy and discectomy. Two weeks postoperative sagittal T2 magnetic resonance imaging (C) shows decompression of cervical spine stenosis.

A 41-year-old male was initially diagnosed with cervical spinal stenosis and a C3-C6 laminectomy and discectomy were performed at an outside institution (Figure 1A-1B). MRI in a neutral position two weeks postoperatively indicated decreased stenosis of the cervical spine (Figure 1C). In the postoperative period, the patient reported particularly concerning episodes of neurological symptoms with new deficits, and increasing levels of tetraparesis.

**Figure 2:**
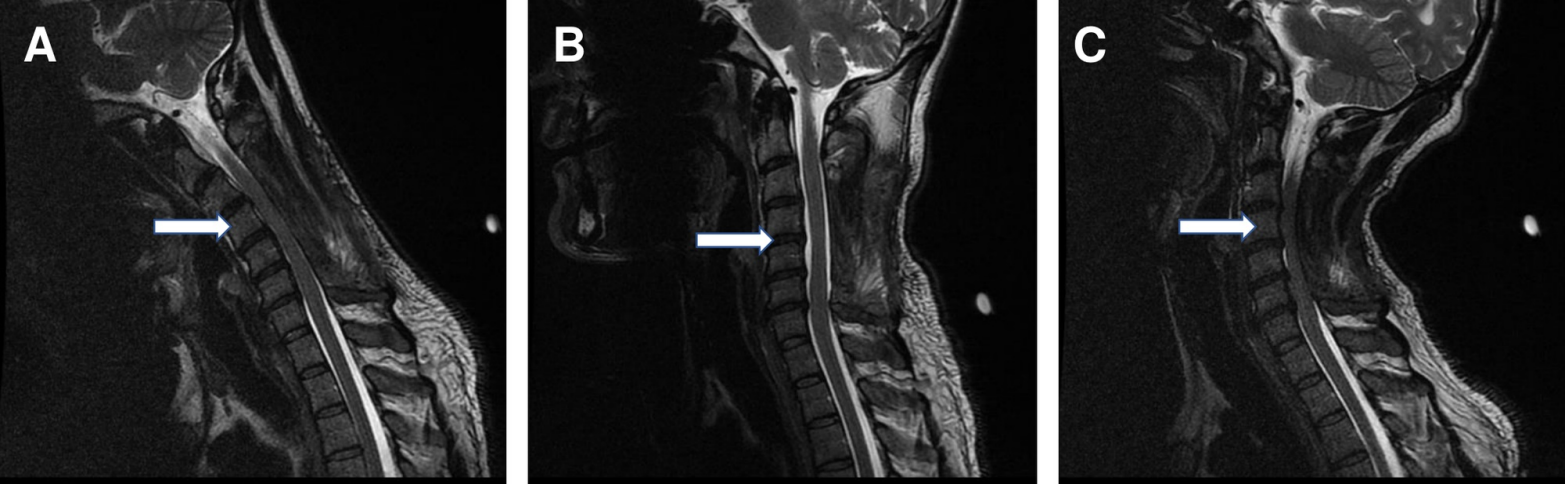
Flexion, Neutral, and Extension Magnetic Resonance Imaging Post-laminectomy Postoperative sagittal T2 magnetic resonance imaging in flexion (A), neutral (B), and extension (C) positions shows minor cervical spinal stenosis in a neutral position with more severe cervical spinal stenosis in flexion and extension positions.

Two months postoperatively, the patient came to our institution and underwent MRI for reevaluation of the cervical spine in three different positions: neutral, flexion, and extension. While neutral MRI did not show any significant stenosis, flexion and extension MRIs were significant for cervical compression due to spondylosis and compression by paraspinal muscles (Figure 2). Compression was quantified by measuring the anteroposterior (AP) diameter of the spinal canal, with more significant compression occurring during extension (Table 1).

**Table 1:** Cervical Spinal Canal Anteroposterior Diameter Measurements Anteroposterior (AP) diameter measurements (mm) of the cervical spinal canal in flexion, neutral, and extension positions

	AP Diameter Measurements (mm)					
MRI Type	C2-C3	C3-C4	C4-C5	C5-C6	C6-C7	C7-T1
Flexion	8.78	7.40	7.30	7.84	10.42	11.79
Neutral	9.36	10.08	10.45	10.07	8.52	11.17
Extension	8.53	4.65	5.09	5.27	8.08	11.09

Surgical management and technique

**Figure 3:**
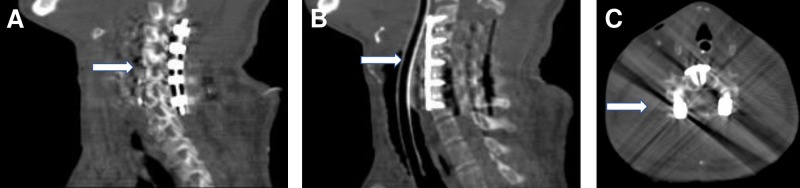
Cervical Spine Computed Tomography After Second Posterior Cervical Decompressive Surgery Postoperative sagittal computed tomography shows bilateral C3-C7 lateral mass screws (A) placed after posterior decompression, and anterior cervical screw-plate instrumentation (B) placed after anterior decompression surgery. Postoperative axial computed tomography (C) shows placement of both posterior and anterior instrumentation.

A reoperative C2-C7 posterior decompression with laminectomies, medial facetectomies, and foraminotomies was performed. Limited undercutting was performed at C2 due to ventral compression visible on the extension at C2. Residual and recurrent compression was encountered and decompressed. Bilateral C3-C7 lateral mass screws were placed, and C2-C7 posterolateral arthrodesis was performed with local autograft and allograft (Figure 3A). Due to clinical and radiographic findings indicating residual nerve root and spinal cord compression, anterior cervical surgery was performed two days later. At surgery via an anterior cervical approach, significant spondylosis and impingement of nerve roots and spinal cord were found at C3-C7. C3-C7 anterior cervical discectomies were performed with posterior osteophytectomies. Structural allograft pieces were contoured and used for the arthrodeses. C3-C7 segmental anterior cervical screw-plate instrumentation was placed (Figure 3B-3C). The patient tolerated the procedure well without complications.

Postoperative course

The patient remained neurologically intact, postoperatively, and had a relatively stable physical exam with occasional numbness and weakness in the right upper extremity. His postoperative course was otherwise uneventful, prompting discharge to rehabilitation. The patient remained stable 6 months postoperatively.

## Discussion

Previous studies on the use of flexion-extension MRI to image the cervical spine found significant variation in spinal cord thickness, spinal cord length, intervertebral disc bulge, ligamentum flavum bulge, and subarachnoid space between flexion and extension positions. Muhle et al. imaged the cervical spine of healthy individuals through 50° of flexion and 30° of extension, finding that, compared to the neutral position, the sagittal cord diameter decreased by up to 14% during flexion and increased by up to 15% during extension [6]. Endo et al. found that the cervical cord length significantly increased during flexion and significantly decreased during extension [18]. For the cervical subarachnoid space, flexion caused ventral narrowing up to 43% and dorsal widening up to 89%; while, extension caused ventral widening up to 9% and dorsal narrowing up to 17% [6]. Chen et al. imaged human cadavers during cervical flexion and extension and measured changes in intervertebral disc bulge and ligamentum flavum bulge. Flexion was associated with a 0.92 mm and 1.12 mm decrease in disc bulge and ligamentum flavum bulge, respectively. Extension was associated with a 0.24 mm and 1.56 mm increase in intervertebral disc bulge and ligamentum flavum bulge, respectively [19]. Thus, cervical flexion is typically associated with decreased cord compression due to cord thinning, widening of overall subarachnoid space, and decreased intervertebral disc and ligamentum flavum bulge. The cervical extension is typically associated with increased cord compression due to opposite trends.

Variation is also found in the determination of severity for cervical spinal stenosis during flexion and extension imaging. Muhle et al. created a four-grade scale for cervical spinal stenosis based on the narrowing of spinal subarachnoid space as well as the physical impingement of the spinal cord, which has been used by multiple studies [5-6, 9, 20]. Ruangchainkom et al. used a modified Muhle grading system scored only by the degree of spinal cord compression [6-7]. Other studies measured the degree of spinal stenosis directly and quantitatively. Lao et al. used a three-grade scale based on the size of cervical disc bulge, with scores increasing above 3 and 5 mm of disc bulge [5]. Dalbayrak et al. measured AP diameter length in the cervical spine during flexion and extension [3]. Sayit et al. measured the length of AP diameter, intervertebral disc bulge, and ligamentum flavum bulge directly during flexion and extension [8]. Imaging and measurements of AP diameter for our case showed significant compression in both flexion and extension, with more severe compression during extension (Table 1, Figure 2). Stenosis would receive the most severe grade on most scales due to significant cord impingement, especially at C3/C4, C4/C5, and C5/C6.

Also, increased compression in our case may be partially explained by the previous laminectomy. Removal of spinal lamina can allow contact and cord compression from posterior paraspinal muscles, which can be seen in our case on flexion and extension MRI before the second decompression surgery (Figure 2B-2C). While increased compression during cervical extension is common, compression during flexion is not. Typically, spinal cord diameter decreases and spinal canal diameter increases during flexion as discussed previously. Muhle et al. found an increase in cord impingement for 27% of patients during cervical extension but only 5% of patients during flexion [6]. Chen et al. found similar results, with cord impingement for 31% of patients during extension but only 3% of patients during flexion [2]. For all previously reported cases of post-laminectomy cord compression by paraspinal muscles with flexion-extension MRI, compression also occurred during extension only [4, 14, 16]. Interestingly, our case shows postoperative compression by paraspinal muscles during flexion and extension due to unknown reasons.

In addition to our case, 13 other cases of postoperative cervical spine stenosis due to paraspinal muscles have been reported [4, 14-17]. Seven out of 14 cases of postoperative compression had no major pathology during neutral position MRI but were diagnosed using extension MRI [4, 14-16]. The other seven cases with post-laminectomy neurological deficits due to compression from paraspinal musculature were identified during a retrospective study by Tu et al. and represented 0.5% of 1309 cervical laminectomy patients [17]. Flexion-extension MRI was not mentioned in the study, making it likely that it was not used for most or all patients. Thus, it is possible that more than 0.5% of patients in Tu et al. would have been diagnosed with postoperative cord compression due to paraspinal musculature if flexion-extension MRI was performed for all patients [17]. More intensive study of a large patient population should be performed to test this hypothesis. If a significant amount of cervical laminectomy patients with postoperative neurological symptoms shows compression only on flexion-extension imaging, an increase in this imaging technique could help improve patient outcomes after cervical spinal decompression surgery.

## Conclusions

Cervical flexion-extension MRI can be used to detect various pathologies not visible on neutral-position MRI. Post-laminectomy cord compression by posterior paraspinal muscles has been reported previously on extension and neutral-position MRI. Post-laminectomy cord compression, in this case, is visible on both flexion and extension MRI but not neutral-position MRI. Increased use of post-laminectomy flexion-extension MRI in cases with postoperative neurological deficits may increase diagnosis of cord compression by paraspinal musculature.
